# Dystroglycan controls dendritic morphogenesis of hippocampal neurons *in vitro*

**DOI:** 10.3389/fncel.2015.00199

**Published:** 2015-05-26

**Authors:** Monika Bijata, Jakub Wlodarczyk, Izabela Figiel

**Affiliations:** Laboratory of Cell Biophysics, Department of Molecular and Cellular Neurobiology, Nencki Institute of Experimental BiologyWarsaw, Poland

**Keywords:** dystroglycan, dendritic arborization, extracellular matrix, MMP-9, Cdc42

## Abstract

Dendritic outgrowth and arborization are important for establishing neural circuit formation. To date, little information exists about the involvement of the extracellular matrix (ECM) and its cellular receptors in these processes. In our studies, we focus on the role of dystroglycan (DG), a cell adhesion molecule that links ECM components to the actin cytoskeleton, in dendritic development and branching. Using a lentiviral vector to deliver short-hairpin RNA (shRNA) that specifically silences DG in cultured hippocampal neurons, we found that DG knockdown exerted an inhibitory effect on dendritic tree growth and arborization. The structural changes were associated with activation of the guanosine triphosphatase Cdc42. The overexpression of DG promoted dendritic length and branching. Furthermore, exposure of the cultures to autoactivating matrix metalloproteinase-9 (aaMMP-9), a β-DG-cleaving protease, decreased the complexity of dendritic arbors. This effect was abolished in neurons that overexpressed a β-DG mutant that was defective in MMP-9-mediated cleavage. Altogether, our results indicate that DG controls dendritic arborization *in vitro* in MMP-9-dependent manner.

## Introduction

Dendritic structure and arborization have a profound impact on the processing of neuronal information (Gulledge et al., 2005; Stuart et al., 2007). The molecular mechanisms that regulate the formation of dendritic trees are precisely controlled. Aberrations in these mechanisms are the basis of several neurodevelopmental disorders, including mental retardation and autism (Kulkarni and Firestein, 2012). Appropriate dendritic branching is driven by numerous extracellular signals, including neurotrophic factors, cell adhesion molecules, and neuronal activity, which lead to changes in cytoskeletal organization (Jan and Jan, 2010; Arikkath, 2012). However, our current understanding of how signal transduction from the extracellular matrix (ECM) controls neuronal morphogenesis is still incomplete.

Dystroglycan (DG) is a cell adhesion receptor composed of α and β subunits that form a link between the ECM and intracellular actin cytoskeleton. DG was identified as a glycan component of the dystrophin glycoprotein complex (DGC) in skeletal muscles (Ervasti and Campbell, 1991), but it is also expressed in the nervous system, epithelia, and endothelia (Durbeej et al., 1998). Extracellular α-DG binds to numerous laminin G domain ligands, including laminins, agrin, and perlecan (Gee et al., 1994; Talts et al., 1999), and presynaptic neurexins in the brain (Sugita et al., 2001). The transmembrane β-DG anchors α-DG to the cell membrane via the N-terminal domain and interacts with the cytoskeletal proteins dystrophin and utrophin via the C-terminal cytoplasmic domain (Ervasti and Campbell, 1991; Barresi and Campbell, 2006). Both subunits undergo posttranslational modifications, such as proteolysis, glycosylation, and phosphorylation (Moore and Winder, 2012). Abnormalities in these processes underlie the pathogenesis of several complex disorders, including dystroglycanopathies and cancer (Singh et al., 2004; Moore and Hewitt, 2009).

Distinct functions of glial and neuronal DG in the brain have been reported (Satz et al., 2010). These studies indicate a crucial role for extracellular α-DG interactions, as the cerebral cortex developed normally in transgenic mice that lacked the DG intracellular domain. β-DG is also known to interact directly with F-actin and thereby may influence actin cytoskeleton remodeling, accompanied by morphological changes (Chen et al., 2003). Moreover, β-DG has been identified as a matrix metalloproteinase-9 (MMP-9) substrate that is digested in response to enhanced neuronal activity (Michaluk et al., 2007). The involvement of MMP-9 in neuronal plasticity and the modulation of dendritic spine morphology has been reported (Michaluk et al., 2011; Dziembowska and Wlodarczyk, 2012; Stawarski et al., 2014). However, the contribution of MMP-9 and its substrates to dendritic morphogenesis is still unclear. Notably, blockade of the proteolytic activity of MMP-9, similarly as DG deletion in neurons, impairs hippocampal long-term potentiation (LTP; Nagy et al., 2006; Satz et al., 2010).

Although β-DG has been shown to be necessary for the formation of filopodia and microvilli-like structures in numerous cell types (Spence et al., 2004a; Batchelor et al., 2007), detailed knowledge of the mechanisms of action of DG in neuronal development is still lacking.

In the present study, we evaluated the effects of the knockdown or overexpression of DG on dendritic arborization in hippocampal neurons *in vitro.* Our results showed that DG deletion simplified dendritic arbor morphology and decreased the total length of dendrites. Conversely, the enhanced neuronal expression of DG resulted in a significant increase in dendritic length and branching. Moreover, we found that treatment of the cultures with autoactivating MMP-9 (aaMMP-9) decreased the complexity of dendritic arbors, and this effect was abolished in neurons that were transfected with a plasmid carrying a β-DG with a mutation in the MMP-9 cleavage site. Furthermore, the DG knockdown-induced simplification of dendritic arbor morphology was dependent on Cdc42 guanosine triphosphatase (GTPase) activity.

## Materials and Methods

### Animals

All of the animal procedures were performed in accordance with the guidelines of the First Local Ethical Committee on Animal Research in Warsaw (permission no. 554/2013), based on the Polish Act on Animal Welfare and other national laws that are in full compliance with EU directives on animal experimentation.

### short-hairpin RNA (shRNA) Constructs and Lentivirus Production

Two short-hairpin RNAs (shRNAs) for DG [SH1 DG (GCUCAUUGCUGGAAUCAUUGC; described previously by Jones et al., 2005) and SH2 DG (UGUCGGCACCUCCAAUUU)] were introduced to a pSilencer (with the U6 promoter) plasmid (Ambion) as double-stranded oligonucleotides. The shRNAs were then subcloned into the pTRIP-PL-WPRE vector together with the SynGFP sequence, which enables the production of lentiviruses that carry shRNA with the simultaneous expression of green fluorescent protein (GFP) driven by the synapsin I promoter. As a control, we used a pTRIP-PL-WPRE vector without shRNA. The lentiviruses were produced in the Laboratory of Cell Engineering, Nencki Institute of Experimental Biology.

### Constructs for Dystroglycan Overexpression

The following expression plasmids were used: DG (α + β) without any tag (OE DG), DG (α + β) fused with GFP (OE DG-GFP), β-DG fused with GFP (OE β-DG-GFP), and β-DG fused with GFP with a mutation in the MMP-9 cleavage site (OE β-DG-MUT-GFP). The neurons were transfected with the aforementioned vectors on the third day *in vitro* (DIV). A red fluorescent protein (RFP)-encoding vector was used to visualize the morphology of transfected cells. All of the overexpressed proteins were under the control of the synapsin I promoter.

The DAG-1 coding sequence was amplified via RT-PCR from total rat RNA using the following DG primers: forward (GCATGTCTGTGGACAACTGGCTACTG) and reverse (CGCGTCGACTTAAGGGGGTACATATGGAGG). The cDNA of the full-length rat DAG1 gene, which encodes DG, was cloned into pDrive vector (pDrive-DG). To generate OE DG, DAG-1 cDNA was subcloned into a vector with the synapsin I promoter (pSyn-GFP). The pSyn-GFP plasmid was cleaved with HpaI and SalI enzymes, and the pDrive-DG plasmid was cleaved with SalI and SnaBI enzymes. OE DG was used to generate OE DG-GFP. We amplified GFP cDNA using the following GFP primers that contained overhangs that introduced NdeI and AseI restriction enzyme sites: forward (CTGATCCATATGTACCCCCTATGGTGAGCAAGGGCGAG) and reverse (GGCCGGATTAATTACTTGTACAGCTCGTCCA). The OE DG plasmid was cleaved with NdeI and AseI enzymes. We checked the obtained constructs for the proper orientation of GFP.

OE DG-GFP was used to generate OE β-DG-GFP. We deleted the cDNA region that encodes α-DG, leaving signaling sequences and regions that encode β-DG using the following primers: forward [TCTATTGTGGTCGAGTGGACCAACA (DG-F-DEL)] and reverse [GGCTTGAGCCACAGCCACAGA (DG-R-DEL)]. The mutation in the cleavage site (OE β-DG-MUT-GFP) was generated by inserting two missense point mutations using the following primers: forward [GCACAGTTCATCCCGTGGCACCACCC (DG-MUT-F)] and reverse [TGCTCGGCAACTGCCAGAGCCCGCCACAG (DG-MUT-R)]. The missense mutation was generated by changing His715 and Leu716 to alanines. The enzymes that were used for cloning were purchased from either New England Biolabs or Thermo Scientific.

### Hippocampal Neuronal Culture and Transfection

Dissociated hippocampal cultures were prepared from postnatal day 0 (P0) Wistar rats as described previously (Michaluk et al., 2011). Cells were plated at a density of 120,000 cells per 18-mm-diameter coverslips (Assistant, Germany) that were coated with 1 mg/ml poly-D-lysine (Sigma) and 2.5 μg/ml laminin (Roche). The cells were kept at 37°C in 5% CO_2_ in a humidified incubator for 1 week. The cells were transfected with Effectene (Qiagen) or Lipofectamine 2000 Reagent (Invitrogen) according to the manufacturer’s protocol on the indicated DIV with the given plasmids. The experiments described below were performed on 8 DIV.

### Cortical Neuronal Culture and Nucleofection

Dissociated cortical cultures were prepared from P0 Wistar rats as described previously (Michaluk et al., 2007). Following isolation, 4 × 10^6^ cortical neurons per nucleofection were centrifuged for 5 min at 800 rcf. The pellet was gently resuspended in 100 μl of nucleofection solution and mixed with 3 μg of the given plasmids. The cell suspension was transferred to a nucleofector cuvette and nucleofected using the O-03 program (Amaxa, Lonza). Immediately after nucleofection, 1 ml of pre-warmed complete growth medium was added to the cuvette. Cells were transferred to six-well plates that contained 1 ml of medium. One hour later, the medium was replaced with 2 ml of prewarmed fresh complete growth medium. The cells were kept at 37°C in 5% CO_2_ in a humidified incubator for 1 week. The experiments were performed on 8 DIV.

For the Cdc42 activation assay, the cortical cell suspension was plated on six-well plates that were covered with 1 mg/ml poly-D-lysine (Sigma) and 2.5 μg/ml laminin (Roche) at a density of 1 × 10^6^ cells per well.

### Lentiviral Treatment of Neuronal Cultures

Primary hippocampal and cortical neurons were infected on 2 DIV with the described lentiviruses. For infection, 1,000 viral particles per cell were used. To determine the silencing effect of the shRNA constructs, the specified amounts of the prepared lentiviral vectors were added to the culture medium according to previously tested ratios.

### Cdc42 Activation Assay

On day 5 *in vitro*, 4 × 10^6^ cortical neurons were homogenized in lysis buffer [25 mM HEPES (pH 7.5), 150 mM NaCl, 1% Non-idet P-40, 10 mM MgCl2, 1 mM ethylenediaminetetraacetic acid (EDTA), and 2% glycerol] and centrifuged at 14,000 × *g* for 10 min. The cell extracts were incubated with GST-PAK-PBD (Cell BioLabs) fusion protein that had been conjugated with glutathione beads at 4°C overnight and washed three times with lysis buffer. GST-PAK-PBD-bound Cdc42 was analyzed by sodium dodecyl sulfate-polyacrylamide gel electrophoresis (SDS-PAGE) and subsequently immunoblotted with Cdc42-specific antibody.

### HEK 293 Culture and Transfection

Human embryonic kidney 293 (HEK 293) cells were grown in Dulbecco’s modified Eagle medium (DMEM; Gibco) that contained 10% fetal bovine serum (FBS; Invitrogen) and 100 U/ml penicillin/streptomycin (Gibco) at 37°C in 5% CO_2_. Transient transfection was performed using Lipofectamine 2000 (Invitrogen) according to the manufacturer’s instructions.

### Treatment with Recombinant MMP-9

The expression of the auto-activating mutant of MMP-9 G100L (aaMMP-9) was evaluated as described previously (Michaluk et al., 2007). Primary hippocampal cultures were treated with 100 ng/ml of aaMMP-9 every day beginning on 3 DIV.

To evaluate Cdc42 activity, primary cortical cultures were treated with 400 ng/ml of aaMMP-9 on day 5 *in vitro* for 10 min. To assess resistance to MMP-9 proteolytic activity, β-DG-MUT-GFP-expressing HEK 293 cells were exposed to aaMMP-9 at a concentration of 400 ng/ml for 3 h.

### Inhibition of Cdc42 Activity

To block Cdc42 activity, the cultures were treated 3 DIV with ZCL 278 (50 μM; Tocris), previously described as a selective Cdc42 inhibitor (Friesland et al., 2013).

### Western Blot

The cells were lysed in RIPA buffer (Sigma) that contained 150 mM NaCl, 1.0% IGEPAL CA-630, 0.5% sodium deoxycholate, 0.1% SDS, and 50 mM Tris, pH 8.0. Protein lysates were then separated by SDS-PAGE and transferred to polyvinylidene difluoride membranes (Immobilon-P, Millipore). The membranes were then blocked with 10% non-fat milk in Tris-buffered saline with 0.1% Tween 20 (TBST). After blocking, the membranes were incubated overnight at 4°C with primary antibodies: anti-β-DG (1:500; ab49515, Abcam), horseradish peroxidase (HRP)-GFP (1:1000; LS-C50850, LifeSpan BioSciences), mouse anti-α-DG (1:1500; 05-593; Millipore), and anti-β-actin (1:10000; A5441, Sigma) diluted in 5% non-fat milk in TBST or anti-GFP (1:1000; MAB3580, Chemicon) and anti-Cdc42 (1:500; 11A11, Cell Signaling) diluted in 5% bovine serum albumin in TBST. The blots were washed three times with TBST followed by 1 h incubation with peroxidase-conjugated secondary antibody, diluted 1:5000 in TBST that contained 5% non-fat milk. After washing, bands were detected using the ECL PRIME chemiluminescence detection system (GE Healthcare).

### Immunocytochemistry

For immunocytochemical studies cultured cells were fixed 8 DIV with 4% paraformaldehyde (PFA) for 10 min at room temperature and permeabilized with 0.1% Triton X-100 in phosphate-buffered saline (PBST) for 10 min. After blocking non-specific binding sites with 10% normal donkey serum (NDS) diluted in PBST for 1 h at room temperature, the cells were incubated with primary antibodies in PBST that contained 5% NDS at 4°C overnight. The following primary antibodies were used: mouse anti-α-DG (1:100; 05-593, Millipore), mouse anti-β-DG (1:50; ab49515, Abcam), rabbit anti-β-DG (1:100; sc-28535, Santa Cruz Biotechnology), and mouse anti-MAP-2 (1:500; M4403, Sigma). After washing with PBS, secondary antibodies conjugated with Alexa Fluor 647 or Alexa Fluor 555 (Molecular Probes, Invitrogen) were applied for 1 h at room temperature. After washing with PBS, the coverslips were mounted in anti-quenching medium (Fluoromount G, Southern Biotechnology Associates, Biozol, Eching, Germany) and subjected to imaging analysis.

The specificity of used antibodies was previously confirmed by other studies (e.g., Michaluk et al., 2007; Pribiag et al., 2014). Indeed, in our hands these antibodies have successfully verified the efficiency of DG knockdown or DG overexpression.

### Image Acquisition and Analysis

To study dendritic morphology, images of neurons were acquired using a Leica TCS SP5 or SP8 confocal microscope with a PL Apo 40×/1.25 NA oil immersion objective. Morphometric analyses were performed using ImageJ with NeuronJ software (Meijering et al., 2004) and the Sholl plugin (Perycz et al., 2011). The axons were excluded during marking tracings for Sholl analysis.

### Statistical Analysis

The data are expressed as mean and SEM. Pair-wise datasets were tested for significant differences using Student’s *t*-test.

## Results

### Knockdown of Dystroglycan Simplifies Dendritic Arbor Morphology

**FIGURE 1:**
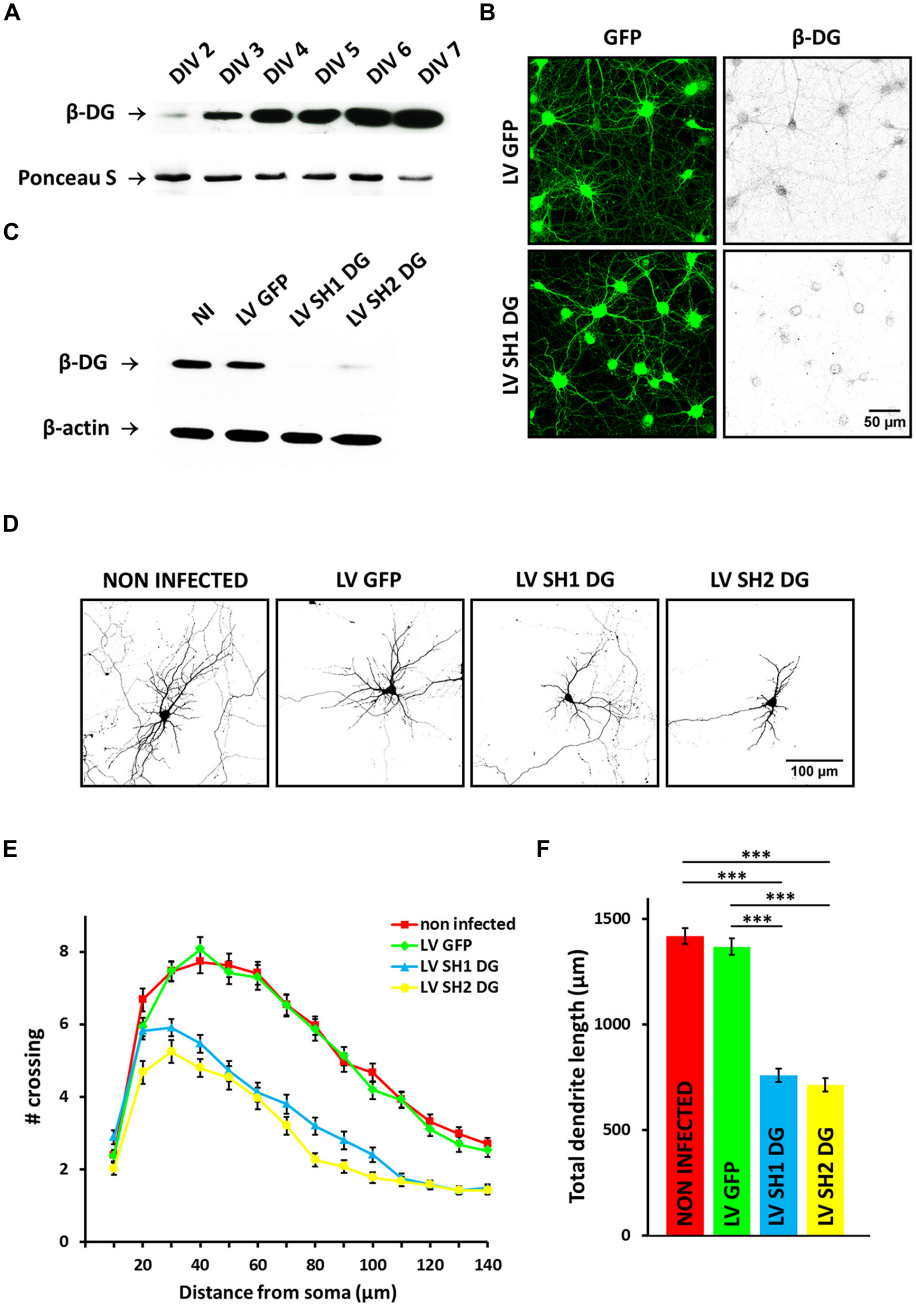
**Knockdown of dystroglycan (DG) impairs dendritic tree development. (A)** Time-course of the DG expression in primary hippocampal cultures – representative western blot analysis is shown. **(B)** Confocal images of hippocampal neurons infected with either empty lentivirus [LV green fluorescent protein (GFP)] or a lentivirus that carries short-hairpin RNA (shRNA) for DG (LV SH1 DG) and immunostained with anti-β-DG antibody. **(C)** Western blot analysis of β-DG expression in protein lysates from non-infected neurons (NI) and neurons infected with LV GFP, LV SH1 DG, or LV SH2 DG. β-actin served as a loading control. **(D)** Representative images of neurons used for the morphometric analysis. The cells were transfected with an RFP-coding vector to visualize the exact morphology. **(E)** Sholl analysis of hippocampal neurons treated as indicated (NI, *n*_cell_ = 109; LV GFP, *n*_cell_ = 85; LV SH1 DG, *n*_cell_ = 87; LV SH2 DG, *n*_cell_ = 51). At distances of 30–140 μm from the cell body, the differences between DG-deficient neurons (LV SH1 DG and LV SH2 DG) and the control groups (NI and LV GFP) were statistically significant (*p* < 0.001). **(F)** Total dendritic length of neurons treated as in **(C)**. Three independent experiments were performed. The data are expressed as mean ± SEM. ^∗∗∗^*p* < 0.001 (Student’s *t*-test).

First we determined the time course of the expression of DG in primary hippocampal cultures. We detected low level of DG on 2 DIV, which was gradually increased up to 7 DIV (**Figure 1A**). To define the possible role of DG in dendritic tree arborization, we decreased its expression using lentiviral shRNA vectors. To verify DG knockdown efficiency, the cells were infected 2 DIV with shRNA1 (LV SH1 DG), shRNA2 (LV SH2 DG), or an empty lentivirus (LV GFP). After 6 days, immunofluorescence staining and Western blot were performed (for detailed kinetic, see Supplementary Figure S1). As shown in **Figure 1B**, neurons that were infected with LV SH1 DG exhibited a reduction of endogenous β-DG expression compared with LV GFP-infected cells. Immunoblotting also revealed efficient β-DG down regulation caused by LV SH1 DG and LV SH2 DG, whereas high levels of β-DG expression were detected in cultures that were infected with LV GFP and control non-infected cultures (**Figure 1C**).

To evaluate the effect of DG silencing on dendritic morphogenesis, 4 days after infection the cultures were additionally transfected with an RFP-encoding vector, which allowed the observation of neuronal morphology. Images for the morphometric analysis were acquired 8 DIV. We selected this time point because intensive dendritogenesis in primary hippocampal neurons occurs during the first week *in vitro*. Representative images of neurons in all of the groups are presented in **Figure 1D**. Infection with LV SH1 DG and LV SH2 DG clearly decreased the complexity of dendritic arbor morphology compared with controls (non-infected cells or cells infected with LV GFP). To precisely evaluate the pattern of dendritic branching, we performed a Sholl analysis (Sholl, 1953), which measures the number of dendrites that cross circles at various radial distances from the cell soma. The results showed that DG silencing induced the shrinkage of dendritic arbors compared with controls, reflected by a downward shift of the Sholl plot (**Figure 1E**). At most of the measured distances (30–140 μm from the cell body), the number of crossings was significantly reduced in DG-deficient neurons (*p* < 0.001). Moreover, the shRNA-mediated knockdown of DG considerably decreased the total dendritic length (**Figure 1F**; non-infected cells: l = 1417 ± 38 μm, *n*_cell_ = 109; LV GFP: l = 1367 ± 38 μm, *n*_cell_ = 85; LV SH1 DG: l = 759 ± 32 μm, *n*_cell_ = 87; LV SH2 DG: l = 713 ± 32 μm, *n*_cell_ = 51; *p* < 0.001). These results may indicate that DG is an important regulator of dendritogenesis in developing primary hippocampal neurons.

### Overexpression of Dystroglycan Promotes Dendritic Growth and Branching

To further confirm the role of DG in dendritic morphogenesis, we developed three plasmid vectors to overexpress DG in primary neurons. The first plasmid encoded DG that was fused with GFP at the C-terminus (OE DG-GFP). However, to exclude the possibility that the observed effect was attributable to disturbances in the C-terminal domain of DG, we also produced a DNA construct without GFP (OE DG). Additionally, to study the function of β-DG alone (without the α subunit) in neuronal morphogenesis, we created a vector that overexpressed β-DG (with a signaling sequence) fused with GFP (OE β-DG-GFP).

**FIGURE 2:**
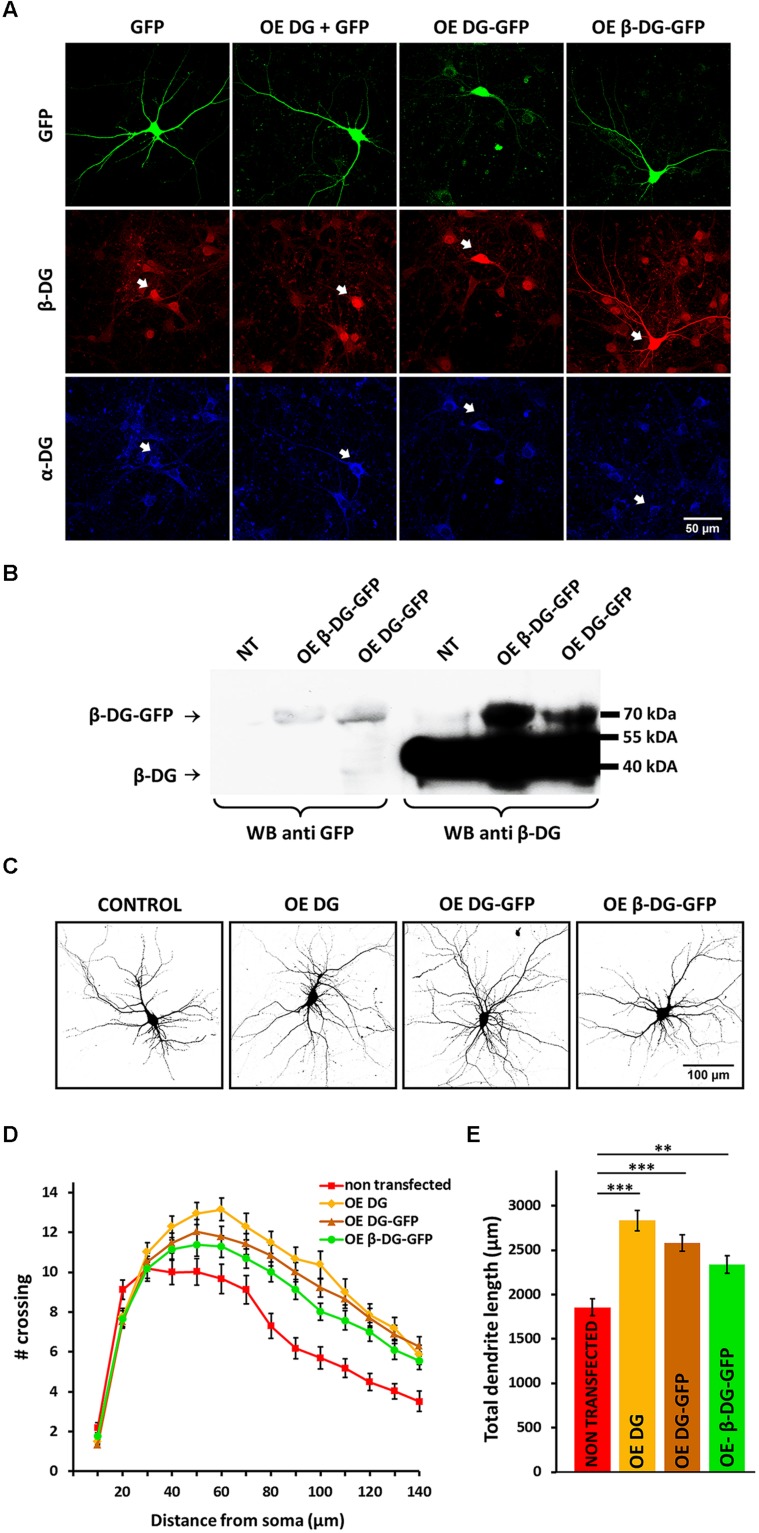
**Overexpression of DG stimulates dendritic outgrowth and arborization. (A)** The upper panel shows confocal images of hippocampal neurons transfected with a GFP-coding vector (GFP) or vectors used for DG overexpression: OE DG (co-transfected with GFP), OE DG-GFP, and OE β-DG-GFP (green). The lower panels show α-DG (blue) and β-DG (red) immunofluorescence staining of cells in the same fields. The arrowheads indicate transfected cells. **(B)** Immunoblot analysis of GFP and β-DG expression in cortical neurons after nucleofection with either OE DG-GFP or OE β-DG-GFP plasmid. Non-transfected cells (NT) served as a control. **(C)** Representative images of neurons used for the morphometric analysis. The cells were co-transfected with an RFP-coding vector to visualize the exact morphology. **(D)** Sholl analysis of hippocampal neurons treated as indicated (NT, *n*_cell_ = 36; OE DG, *n*_cell_ = 40; OE DG-GFP, *n*_cell_ = 57; OE β-DG-GFP, *n*_cell_ = 56). At distances of 80–120 μm from the cell body, the differences between DG-overexpressed neurons (OE DG, OE DG-GFP, and OE β-DG-GFP) and the control group (NT) were statistically significant (*p* < 0.001). **(E)** Total dendritic length for neurons treated as in **(C)**. Three independent experiments were performed. The data are expressed as mean ± SEM. ^∗∗^*p* < 0.01, ^∗∗∗^*p* < 0.001 (Student’s *t*-test).

To verify the effectiveness of the aforementioned constructs, we first evaluated the expression of α- and β-DG by immunofluorescence and Western blot. In neurons that were transfected with the OE DG, OE DG-GFP, or OE β-DG-GFP vector, we observed enhanced β-DG immunostaining compared with neighboring cells (**Figure 2A**). At the same time we noted increased immunoreactivity of α-DG only in neurons transfected with the OE DG or OE DG-GFP plasmid (**Figure 2A**). For the Western blot analysis, we used nucleofection technology (Amaxa, Lonza) to obtain high-efficiency transfection. The immunoblotting of protein lysates with anti-β-DG and anti-GFP antibodies revealed a band of approximately 70 kDa, which corresponds to β-DG fused with GFP (**Figure 2B**). The level of overexpression was lower than the endogenous levels of β-DG, however by using synapsin promoter we obtained increased expression of β-DG exclusively in neurons, while endogenously it is also expressed in glial cells present in hippocampal cultures. In addition, we observed increased level of α-DG in HEK 293 cells transfected with OE DG-GFP vector (see Supplementary Figure S2). These results confirmed that we were able to obtain DG overexpression in transfected cells, and this DG overexpression was properly ascribed to the α and β subunits.

To assess the effect of DG overexpression on dendritic arbor morphology, the cultures were transfected with the OE DG, OE DG-GFP, or OE β-DG-GFP vector and co-transfected with an RFP-coding vector 3 DIV. Images for the Sholl analysis were acquired 8 DIV. As shown in **Figure 2C**, neurons that overexpressed DG exhibited enhanced dendritic arborization, regardless of the presence of the GFP tag in the construct. This fact indicates that the presence of GFP likely did not interfere with the C-terminal-mediated function of DG.

The morphometric analysis revealed an upward shift of the Sholl plot in neurons that were transfected with the OE DG, OE DG-GFP, or OE β-DG-GFP vector compared with non-transfected cells (**Figure 2D**), indicating an increase in the complexity of dendritic trees. At most of the measured distances (80–120 μm from the cell body), the number of crossings significantly increased in neurons that overexpressed DG or β-DG (*p* < 0.001). The overexpression of DG also affected dendritic outgrowth (**Figure 2E**). The total dendritic length increased in neurons that were transfected with OE vectors compared with non-transfected cells (OE DG: l = 2835 ± 114 μm, *n*_cell_ = 40, *p* < 0.001; OE DG-GFP: l = 2582 ± 92 μm, *n*_cell_ = 57, *p* < 0.001; OE β-DG-GFP: l = 2338 ± 97 μm, *n*_cell_ = 56, *p* < 0.002; non-transfected cells: l = 1856 ± 92 μm, *n*_cell_ = 36).

The enhancing effect on dendritic trees was observed only in cells that were transfected 3 DIV. When transfection was performed 5 DIV, these morphological changes were not evident (data not shown). This observation indicates that DG is particularly necessary during the early stages of dendritic development. Interestingly, the overexpression of β-DG alone (without α-DG) fused with GFP evoked a very similar effect on dendritic arbors as overexpressed full-length DG. These data suggest an important role for β-DG in neuronal development.

### MMP-9 Inhibits Dendritic Growth and Branching

**FIGURE 3:**
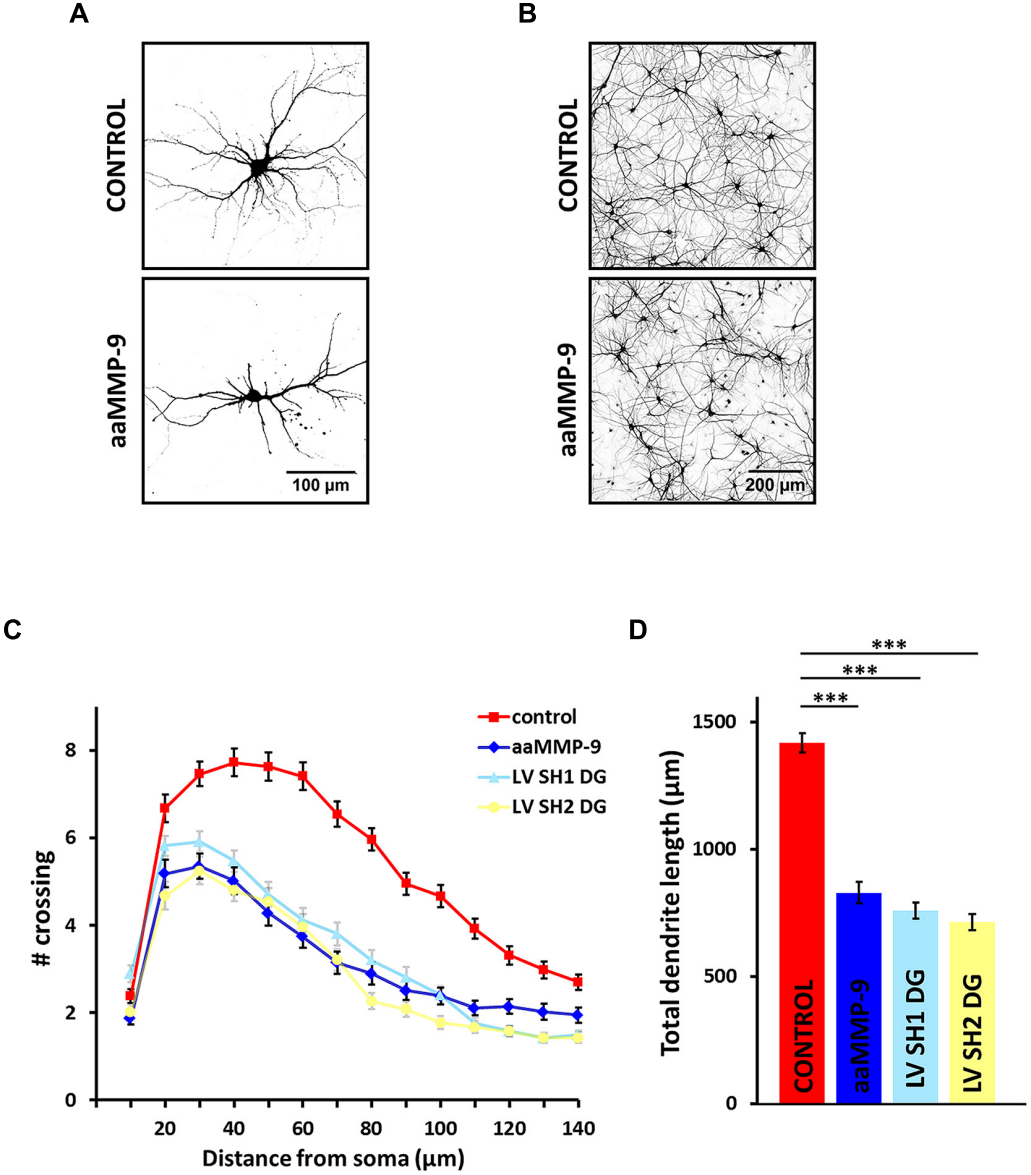
**Matrix metalloproteinase-9 (MMP-9) activity inhibits dendritic growth and arborization. (A)** Representative images of hippocampal neurons from control culture and cultures treated with autoactivating MMP-9 (aaMMP-9). The cells were transfected with an RFP-coding vector to visualize the exact morphology. **(B)** Representative images of hippocampal cultures treated as in **(A)** following immunofluorescence staining with anti-MAP2 antibody. **(C)** Sholl analysis of hippocampal neurons exposed to aaMMP-9 (*n*_cell_ = 55) vs. control (*n*_cell_ = 36) and neurons infected with either LV SH1 DG (*n*_cell_ = 87) or LV SH2 DG (*n*_cell_ = 51). At distances of 20–130 μm from the cell body, the differences between aaMMP-9-treated neurons and the control group were statistically significant (*p* < 0.001). At distances of 10–90 μm from the cell body, no statistically significant differences were found between aaMMP-9-treated neurons and neurons infected with LV SH1 DG or LV SH2 DG. **(D)** Total dendritic length for neurons treated as in **(C)**. All of the experiments were performed in triplicate. The data are expressed as mean ± SEM. ^∗∗∗^*p* < 0.001 (Student’s *t*-test).

β-dystroglycan has been previously shown to be proteolytically processed by MMP-9 in activated neurons (Michaluk et al., 2007). Thus, we decided to examine the effect of exogenous MMP-9 on dendritic arborization. We treated primary hippocampal cultures with recombinant aaMMP-9 once per day beginning 3 DIV. As shown in **Figure 3A**, 5-day exposure to aaMMP-9 reduced dendritic tree arborization compared with control untreated neurons. Similarly, immunostaining against microtubule-associated protein 2 (MAP2) showed decreased complexity of the neural network in aaMMP-9-treated cultures (**Figure 3B**).

The morphometric analysis revealed that enhanced MMP-9 activity evoked the shrinkage of dendritic arbors compared with untreated controls, reflected by a downward shift of the plot (**Figure 3C**). At most of the measured distances (20–130 μm from the cell body) the number of crossings was significantly reduced in aaMMP-9-treated neurons (*p* < 0.001). MMP-9 activity also decreased the total dendritic length (**Figure 3C**; untreated cells: l = 1417 ± 38 μm, *n*_cell_ = 109; aaMMP-9: l = 829 ± 43 μm, *n*_cell_ = 55; *p* < 0.001). Interestingly, the morphology of aaMMP-9-treated neurons was very similar to the morphology of DG-deficient neurons. At distances of 10–90 μm from the cell body, no statistically significant differences were found between aaMMP-9-treated neurons and neurons that were infected with LV SH1 DG or LV SH2 DG. These groups were also very comparable with regard to total dendritic length, which was approximately 50% less than in controls (**Figure 3D**). These results may indicate that MMP-9 is also an important regulator of dendritogenesis and suggest that DG and MMP-9 cooperate in regulating the morphology of dendritic arbors.

### MMP-9-Mediated Cleavage of β-DG is Involved in Neuritogenesis

**FIGURE 4:**
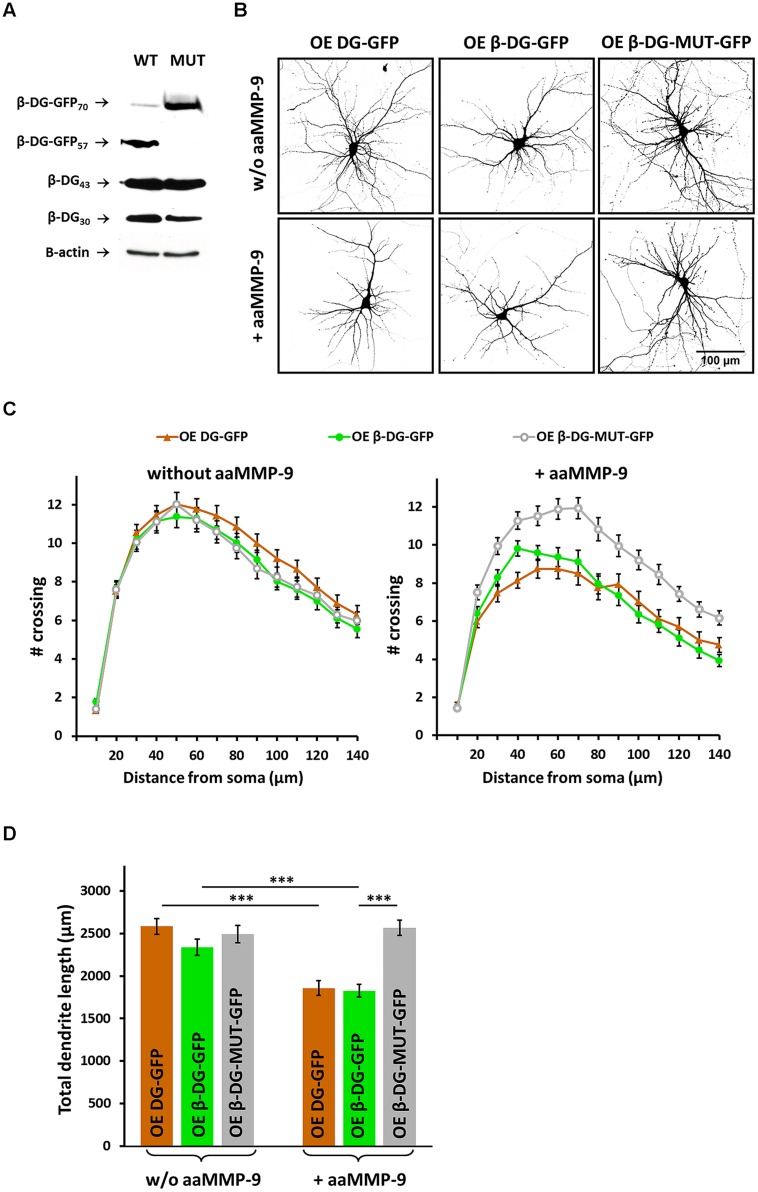
**Matrix metalloproteinase-9 dependent cleavage of β-DG is important for dendritic development. (A)** Immunoblot analysis of β-DG and GFP expression in protein lysates from HEK 293 cells transfected with either OE β-DG-GFP (WT) or OE β-DG-MUT-GFP (MUT) plasmids and then treated with aaMMP-9. β-actin served as a loading control. **(B)** Representative images of neurons used for the morphometric analysis. The cells were co-transfected with an RFP-coding vector to visualize the exact morphology. **(C)** Sholl analysis of hippocampal neurons treated as indicated (*n*_cell_ = 50 ± 4). In control cultures (without aaMMP-9), no statistically significant differences were found between DG-overexpressed neurons (transfected with either OE DG-GFP or OE β-DG-GFP) and neurons transfected with the OE β-DG-MUT-GFP vector. In cultures treated with aaMMP-9, at distances of 30–140 μm from the cell body, the differences between DG-overexpressed neurons (OE DG-GFP and OE β-DG-GFP) and neurons that overexpressed mutated β-DG (OE β-DG-MUT-GFP) were statistically significant (*p* < 0.02). **(D)** Total dendritic length for neurons treated as indicated. All of the experiments were performed in triplicate. The data are expressed as mean ± SEM. ^∗∗∗^*p* < 0.001 (Student’s *t*-test).

We found that the silencing of DG and enhanced activity of MMP-9 exert similar effects on dendritic morphology. To test whether this similarity arises from the fact that MMP-9 inactivates DG by proteolytic processing, we modified the protease cleavage site in the OE β-DG-GFP vector using site-directed mutagenesis. The OE β-DG-MUT-GFP vector was introduced into HEK 293 cells, and its resistance to cleavage by MMP-9 was determined. We performed Western blot using an anti-GFP antibody to verify the cleavage of overexpressed β-DG and anti-β-DG to verify the cleavage of endogenous β-DG. As shown in **Figure 4A**, the mutation prevented the cleavage of β-DG by MMP-9 compared with wild type β-DG.

We then attempted to determine whether chronic MMP-9 activity can prevent the enhancement of dendritogenesis that is caused by DG overexpression. Neurons were transfected with OE DG-GFP, OE β-DG-GFP, or OE β-DG-MUT-GFP and treated once per day with aaMMP-9 beginning 3 DIV. As shown in **Figure 4B**, aaMMP-9 treatment disrupted dendritogenesis in neurons that overexpressed DG or β-DG but did not influence the morphology of neurons that were transfected with OE β-DG-MUT-GFP.

The results of the Sholl analysis showed that treatment with aaMMP-9 induced the shrinkage of dendritic arbors in neurons that were transfected with either OE DG-GFP or OE β-DG-GFP, whereas we did not observe any differences in neurons that were transfected with OE β-DG-MUT-GFP after aaMMP-9 stimulation (**Figure 4C**). At most of the measured distances (20–140 μm from the cell body), the number of crossings in OE DG-GFP- or OE β-DG-GFP-transfected neurons significantly reduced upon exposure to aaMMP-9 (*p* < 0.02 and 0.04, respectively). The number of crossings in OE β-DG-MUT-GFP-transfected neurons did not significantly change after stimulation with aaMMP-9.

We also evaluated the effect of aaMMP-9 treatment on total dendritic length. In neurons that overexpressed DG-GFP or β-DG-GFP, daily stimulation with aaMMP decreased this parameter (from l = 2582 ± 92 μm, *n*_cell_ = 57 for OE DG-GFP to l = 1856 ± 86 μm, *n*_cell_ = 38 for OE DG-GFP + aaMMP-9, *p* < 0.001; from l = 2338 ± 97 μm, *n*_cell_ = 56 for OE β-DG-GFP to l = 1825 ± 75 μm, *n*_cell_ = 50 for OE β-DG-GFP + aaMMP-9, *p* < 0.001; **Figure 4D**). In contrast, the total dendritic length was unchanged in OE β-DG-MUT-GFP-transfected neurons upon aaMMP-9 stimulation (from l = 2494 ± 102 μm, *n*_cell_ = 46 for OE β-DG-MUT-GFP to l = 2566 ± 90 μm, *n*_cell_ = 47 for OE β-DG-MUT-GFP + aaMMP-9, *p* = 0.39; **Figure 4D**).

We also compared the effect of aaMMP-9 on OE β-DG-GFP-transfected neurons with neurons that expressed OE β-DG-MUT-GFP. We observed a significantly lower number of crossings (50–140 μm from the cell body, *p* < 0.002) and a decrease in total dendritic length (*p* < 0.001) in cells that were transfected with wild type β-DG-GFP. These results indicate that the MMP-9-mediated cleavage of β-DG is important for morphological changes that accompany neuritogenesis.

### Morphological Effects of DG Knockdown and MMP-9 Treatment are Rescued by the Inhibition of Cdc42 Activity

**FIGURE 5:**
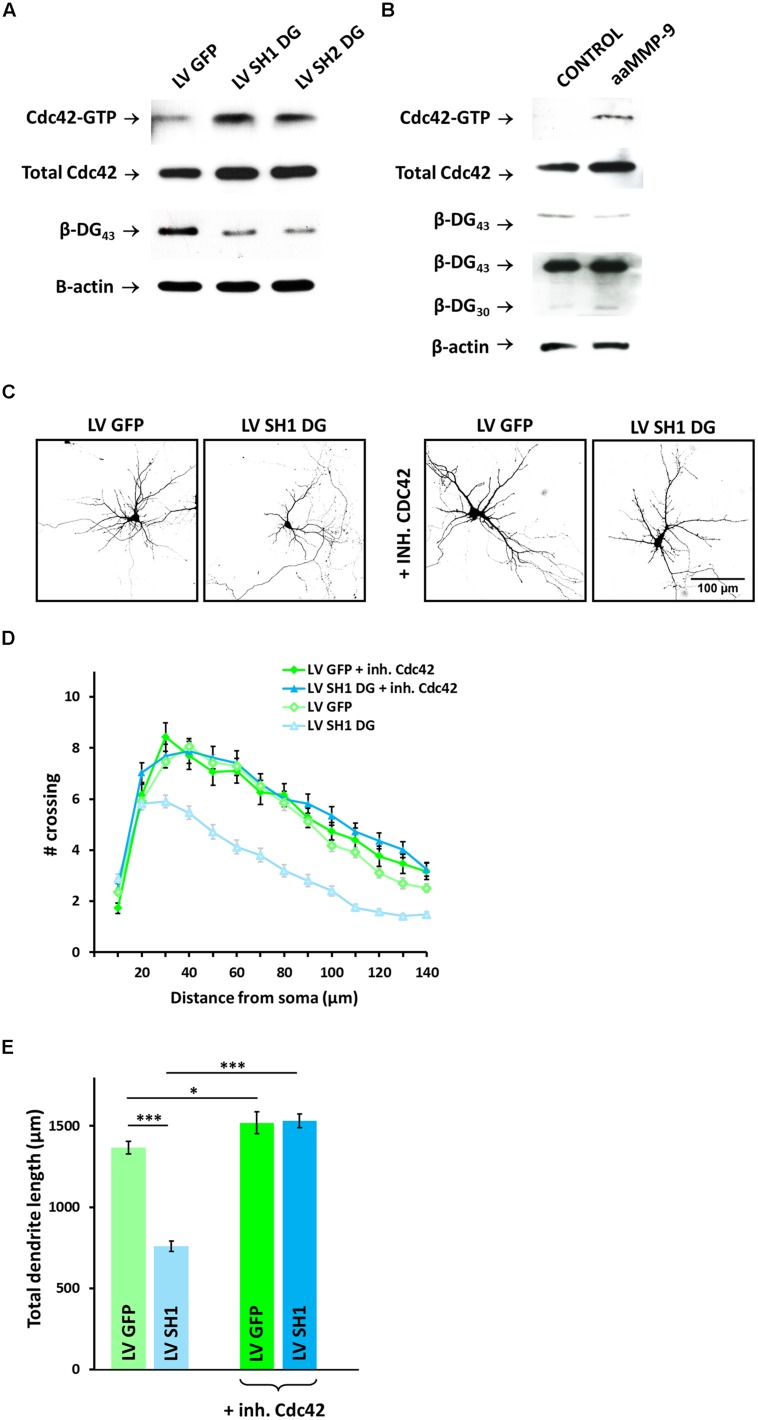
**Cdc42 activity contributes to dendritic atrophy caused by DG knockdown or MMP-9 treatment. (A)** Western blot analysis of active Cdc42 (Cdc42-GTP) and total Cdc42 in cortical neurons infected with LV GFP, LV SH1 DG, or LV SH2 DG. Silencing efficiency was checked by immunoblotting with anti-β-DG antibody. β-actin was used as a loading control. **(B)** Western blot analysis of active Cdc42 (Cdc42-GTP) and total Cdc42 in control and aaMMP-9-treated cortical neurons. Immunoblotting with anti-β-DG served as a control for the proteolytic activity of MMP-9. β-actin was used as the loading control. **(C)** Representative images of neurons used for the morphometric analysis. The cells were transfected with an RFP-coding vector to visualize the exact morphology. **(D)** Sholl analysis of hippocampal neurons treated as indicated (LV GFP, *n*_cell_ = 85; LV SH1 DG, *n*_cell_ = 87; LV GFP + Cdc42 inhibitor, *n*_cell_ = 28; LV SH1 DG + Cdc42 inhibitor, *n*_cell_ = 51). At distances of 20–140 μm from the cell body, the differences between DG-deficient neurons that were treated with the Cdc42 inhibitor and DG-deficient neurons that were not treated with the Cdc42 inhibitor were statistically significant (*p* < 0.001). **(E)** Total dendritic length for neurons treated as indicated. Three independent experiments were performed. The data are expressed as mean ± SEM. ^∗^*p* < 0.05, ^∗∗∗^*p* < 0.001 (Student’s *t*-test).

Cdc42 GTPase is well known to play an important role in the growth and branching of axons and dendrites. DG has been shown to cooperate with Cdc42 to induce the formation of filopodia structures. Therefore, we examined whether DG knockdown influences the activity of Cdc42 in cultured cortical neurons using a pull-down assay with GST-PAK-PBD. We observed an increase in the level of GTP-bound Cdc42 (i.e., active Cdc42) in SH1 DG- and SH2 DG-infected neurons compared with control untreated cells (**Figure 5A**). The enhanced activation of Cdc42 GTPase was associated with a reduction of the complexity of dendritic trees since the similar Cdc42 activation was also observed in cultures that were exposed to aaMMP-9 (**Figure 5B**).

We next determined whether Cdc42 inhibition rescues dendritogenesis in DG-deficient neurons. LV SH1 DG-infected hippocampal neurons were treated with 50 μM of the selective Cdc42 inhibitor ZCL 278 (Friesland et al., 2013). As shown in **Figure 5C**, the disruption of dendritogenesis was rescued by blocking Cdc42 activity. Moreover, we did not observe any significant differences in dendritic arborization in LV GFP-infected neurons evoked by treatment with the Cdc42 inhibitor.

The Sholl analysis revealed similar upregulation of dendritic tree complexity in LV GFP-infected neurons, regardless of ZCL 278 treatment, and LV SH1 DG-infected cells that were exposed to ZCL 278 compared with DG-deficient neurons that were only infected with LV SH1 DG. The number of crossings was significantly higher at most of the measured distances (20–140 μm from the cell body; *p* < 0.001; **Figure 5D**).

Similarly, a high degree of rescue was observed with regard to total dendritic length (from l = 759 ± 32 μm, *n*_cell_ = 87 for SH1 DG to l = 1531 ± 40 μm, *n*_cell_ = 51 for SH1 DG + ZCL 278, *p* < 0.001; **Figure 5E**). This parameter was only slightly increased upon Cdc42 inhibition in LV GFP-infected cells.

These results suggest that DG plays a crucial role in the process of neuritogenesis through the inhibition of Cdc42 activity. Furthermore, the enhancement of proteolytic MMP-9 activity may cause DG inactivation through cleavage, leading to the disinhibition of Cdc42 and impairments in dendritic arbor development.

## Discussion

Dystroglycan is a major ECM receptor that is expressed in various tissues, including the central nervous system (Durbeej et al., 1998; Zaccaria et al., 2001). Deletion of the gene that encodes DG (*Dag1*) causes early embryonic lethality in mice, indicating its important role during mammalian development (Williamson et al., 1997). Previous studies demonstrated that DG expressed on glial cells is responsible for basement membrane formation, whereas neuronal DG facilitates hippocampal LTP, one of several phenomena that underlie synaptic plasticity (Satz et al., 2010). The number and pattern of synapses that are received by each neuron are well known to be determined by dendritic outgrowth and arborization (Koleske, 2013). However, unknown is whether DG affects dendritic development, which is crucial in the formation of functional neural networks.

The present study provides evidence that DG plays an important role in early stages of neuritogenesis in cultured hippocampal neurons. We detected a high level of DG expression at 1 week in cultures. These results contradict previous research published by Lévi et al. (2002) showing a very weak expression of DG during maturation of hippocampal neurons *in vitro*. These discrepancies may be due to differences in the models used in both studies. We found that the lentiviral-mediated shRNA knockdown of DG reduced dendritic growth and branching. In contrast, the overexpression of either full-length DG or β-DG alone had the opposite effect (i.e., the promotion of dendritic tree development). Furthermore, our results indicated that the observed disturbances in dendritic arborization might be caused by the MMP-9-mediated proteolysis of DG and are associated with Cdc42 GTPase activation.

Neuron–ECM interactions are considered essential for proper dendritic development and function (Myers et al., 2011; Skupien et al., 2014; Thalhammer and Cingolani, 2014). DG is a high-affinity receptor for several ECM components, including laminin (Ervasti and Campbell, 1991, 1993). The importance of the DG-laminin interaction is supported by the fact that mice with astrocyte-specific DG deletion exhibit discontinuities in pial surface basal lamina (Moore et al., 2002). Since in the present study we used cells grown on laminin, it would be interesting to check whether the simplification of dendritic arbors evoked by DG silencing is attributable to the weakening of laminin binding.

We further found that the overexpression of OE DG, OE DG-GFP, or OE β-DG-GFP promoted hippocampal dendritic development. Interestingly, the overexpression of β-DG caused the same effect on dendritic arborization as overexpression of full-length DG that contained the α and β subunits. This may indicate the important role of downstream signaling mediated by β-DG. DG has been reported to be a scaffold protein that interacts with components of the ERK-MAP kinase cascade (Spence et al., 2004b; Moore and Winder, 2010). Moreover, the cytoplasmic tail of β-DG has been shown to interact with utrophin, an actin-binding protein, and directly with the actin cytoskeleton (Chen et al., 2003).

We observed a pronounced effect of DG on dendritic maturation. We tested whether the MMP-9-mediated cleavage of DG is functionally involved in this process. We found that daily treatment of hippocampal cultures with aaMMP-9 for 5 days inhibited dendritic growth and branching. Interestingly, this effect coincided with the effect induced by infection with LV SH DG. Given the results that indicated that MMP-9 interferes with proper dendritic arbor formation, we next considered how β-DG mutation in the MMP-9 cleavage site might affect dendritogenesis. We found that the overexpression of β-DG-MUT-GFP abolished the growth-inhibiting effect of MMP-9 on dendritic trees. Matrix metalloproteinases were once believed to regulate neurite extension by simply degrading the surrounding ECM to create a passage for process outgrowth (Ethell and Ethell, 2007; Sarig-Nadir and Seliktar, 2010). However, more recent *in vitro* and *in vivo* studies suggest that proteolytic cleavage is directed toward specific ligands and receptors to regulate neuronal development and regeneration (Fujioka et al., 2012). In the present study, we found that MMP-9 may act as a regulator of dendritic arbor maturation, and the digestion of DG is important for this activity.

Actin reorganization that accompanies morphological changes is generally known to be regulated by Rho-family small GTPases, such as Rho, Rac, and Cdc42. Their activity is strictly controlled by multiple guanine nucleotide exchange factors (GEFs), guanine nucleotide dissociation inhibitors (GDIs), and GTPase-activating proteins (GAPs). DG was previously shown to bind the cytoskeletal adapter protein ezrin and form a complex with a Rho GEF that is responsible for activating Cdc42 and thus causing filopodia formation in fibroblasts (Batchelor et al., 2007). In the present study, we examined the expression of active Cdc42 in LV SH DG-infected neurons and neurons treated with aaMMP-9. In both cases, we observed an increase in Cdc42 activity. This result was surprising because in most studies published to date, Cdc42 inhibition resulted in significant simplification of dendritic trees in many types of neuron (Govek et al., 2005; Negishi and Katoh, 2005). However, the functional outcome of the activity of GTPases must be considered within the context of additional considerations, such as the temporal control of activation and cell type. Notably, the signaling pathways that employ Rho GTPases are highly regulated and can produce different cellular responses under different cellular conditions. For example, both constitutively active Cdc42 and Rac1 that is expressed in *Drosophila* giant fiber neurons inhibit neurite outgrowth (Allen et al., 2000). Moreover, our data are consistent with the observation that the suppression of Cdc42 signaling during mouse cortical development is required to promote the branching of dendritic trees (Rosário et al., 2012). In the same paper, the authors showed that mutations in Cdc42 GAP (NOMA-GAP) resulted in hyperactive Cdc42 and simplified dendritic branches. The complex role of Cdc42 in dendritogenesis has recently been reviewed (Simó and Cooper, 2012). One unresolved issue involves the DG-dependent intracellular signaling pathways that regulate Cdc42 and cytoskeletal rearrangement.

Altogether, we established that DG is important for dendritic outgrowth and arborization in primary hippocampal neurons. Further studies are required to elucidate the signaling mechanisms of DG action. However, our findings indicate the involvement of MMP-9-mediated proteolytic cleavage of the β-DG extracellular domain and intracellular activation of Cdc42 GTPase. These results may provide insights into the role of interactions between cell adhesion receptors and the ECM in neuritogenesis and synaptic plasticity and open the way to *in vivo* studies that may help to understand the molecular bases of several neurological disorders, including mental retardation associated with dystroglycanopathies.

## Author Contributions

Conceived the study: MB, IF. Experimental design: MB, IF. Performed the experiments: MB, IF. Analyzed the data: MB, IF. Contributed reagents/materials/analysis tools: MB, JW, IF. Wrote the paper: MB, JW, IF.

## Conflict of Interest Statement

The authors declare that the research was conducted in the absence of any commercial or financial relationships that could be construed as a potential conflict of interest.
