# Supplementary material for: Dystroglycan controls dendritic morphogenesis of hippocampal neurons in vitro
Source: Front Cell Neurosci. 2015 May 26;9:199. doi: 10.3389/fncel.2015.00199 (PMC4443029; doi:10.3389/fncel.2015.00199)
Supplement: Supplementary file 1 [file Data_Sheet_1.PDF]

Supplementary Material

Dystroglycan controls dendritic morphogenesis of hippocampal neurons *in vitro*

Monika Bijata, Jakub Wlodarczyk, Izabela Figiel*

Laboratory of Cell Biophysics, Department of Molecular and Cellular Neurobiology, Nencki Institute, Warsaw, Poland

* **Correspondence:** Izabela Figiel, Laboratory of Cell Biophysics, Department of Molecular and Cellular Neurobiology, Nencki Institute, Str. Pasteura 3, Warsaw, 02-093, Poland, i.figiel@nencki.gov.pl

Supplementary Figures

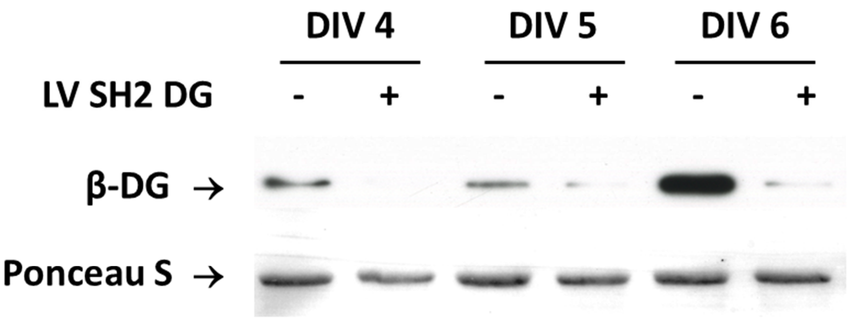

Figure S1. Detailed kinetic of dystroglycan silencing in primary hippocampal cultures. Western blot analysis of β-DG expression in protein lysates from neurons infected on 2 DIV with LV SH2 DG. Efficient knock-down of DG was detected 2 days following infection.

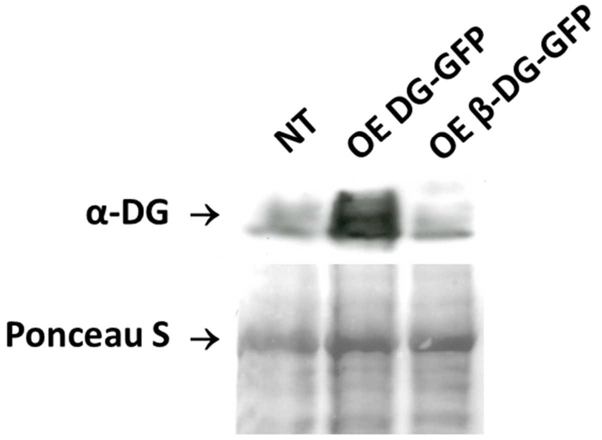

Figure S2. Efficient overexpression of α-dystroglycan in HEK 293 cells. Cell were lysed 3 days following transfection with either OE DG-GFP or OE β-DG-DG plasmids and western blot was performed with anti-α-DG antibody. NT- protein extracts from non-transfected cells.
